# Linking Intradialytic Blood Volume Dynamics to Extracellular Fluid Status: Toward Personalized Fluid Assessment in Hemodialysis

**DOI:** 10.3390/jcm14207188

**Published:** 2025-10-12

**Authors:** Martin Russwurm, Marvin Braun, Julia Menne, Lara Ploeger, Marc Miran, Fabian Max, Lotte Dahmen, Joachim Hoyer, Johannes Wild

**Affiliations:** 1 Department of Internal Medicine and Nephrology, University Hospital Marburg, Baldingerstrasse 1, 35043 Marburg, Germany; 2 Institute of Pharmacology, University Marburg, 35043 Marburg, Germany

**Keywords:** hemodialysis, relative blood volume, bioimpedance spectroscopy, extracellular water, ultrafiltration, fluid management, personalized medicine

## Abstract

**Background**: Accurate assessment of volume status remains a central challenge in hemodialysis (HD). Although bioimpedance spectroscopy (BIS) can quantify fluid compartments, it is time-consuming and requires a lot of personnel. Modern HD machines provide continuous relative blood volume (RBV) monitoring. We examined whether intradialytic RBV dynamics reflect pre-dialysis extracellular fluid (ECW) status to inform personalized fluid management. **Methods**: In an ancillary, monocentric, prospective study of the SkInDialysis trial (DRKS00036332), 11 maintenance-HD patients underwent three standardized dialysis sessions with simultaneous measurement of RBV and BIS. BIS was performed at five time points per session (pre-HD; 20, 80, and 160 min after the start of HD; and post-HD). Ultrafiltration (UF), RBV, total body water (TBW), ECW, and intracellular water (ICW) were recorded. **Results**: Mean total UF was 2809 ± 894 mL/session. RBV declined to 94.7 ± 3.1% at 20 min and to 87.6 ± 5.5% by the end of the session. TBW decreased by 2.9 ± 2.7%, driven by ECW reduction (−3.15 ± 2.9%) over ICW (−1.1 ± 1.65%). Cumulative UF correlated with declines in TBW (R^2^ = 0.18; *p* = 0.02) and ECW (R^2^ = 0.23; *p* = 0.01) and more modestly with ICW (R^2^ = 0.16; *p* = 0.04). In contrast, ΔRBV (pre- vs. post-HD) did not correlate with UF, weight loss, or compartmental water changes. Early steady-state RBV at 80 min correlated with pre-HD ECW (R^2^ = 0.19; *p* = 0.02) and more strongly with the pre-HD ECW/ICW ratio (R^2^ = 0.34; *p* = 0.001). **Conclusions**: In this small, repeated-measures cohort, absolute early steady state RBV levels were associated with pre-dialysis ECW and the ECW/ICW ratio, whereas RBV change (ΔRBV) did not track absolute fluid removal. Our data support a time-anchored RBV level as a pragmatic, device-embedded indicator of the pre-dialysis extracellular reservoir.

## 1. Introduction

Personalized or precision medicine are two popular buzzwords in the context of current therapeutic attempts [1,2]. These concepts also apply to hemodialysis (HD) patients, with the key goal being to tailor treatment to each individual patient as much as possible. This involves internationally discussed trends such as incremental hemodialysis [3,4] and the possible advantages of hemodiafiltration [5] over hemodialysis, as well as the seemingly trivial question of how to best assess fluid balance. Assessing target weight and thus optimizing fluid removal during dialysis, or detecting and quantifying volume excess, has remained a challenge since the early days of HD until today [6,7,8] and remains a major concern in daily dialysis practice [9]. Accurate fluid assessment beforehand is required to achieve the right balance between sufficient ultrafiltration (UF) to prevent overhydration and maintaining hemodynamic stability despite volume depletion. The main pillars for volume assessment remain medical history and physical examination, primarily because no diagnostic equipment and thus no additional costs are required that way [7]. Furthermore, this approach without additional instrument-based diagnostics ensures efficient initiation of hemodialysis, reducing transition times between sessions and thereby optimizing resource utilization. However, substantial shortcomings of clinical assessments regarding correct recognition of hypervolemia and hypovolemia are evident [10,11,12]. Alongside purely clinical approaches, instrumental methods for assessing fluid status in HD patients have been proposed for many years, yet their impact on mortality remains controversial [13]. For example, bedside ultrasound diagnostics has become significantly more important and well-studied in almost every discipline in recent years. The trend towards point-of-care ultrasound (POCUS) has not stopped at HD patients either [14]. Nevertheless, the value of POCUS, especially lung POCUS, is confined to diagnosing hypervolemia, whereas hypovolemia cannot be assessed. As a fast and non-invasive procedure, there is evidence that HD patients might benefit from ultrasound diagnostics for fluid assessment [15,16,17,18]. However, the major downside of any application of ultrasound diagnostics is its considerable variability between examiners. In theory, a preferable apparative measure of volume status provides objective, reliable results. 

Bioimpedance technologies are validated for the assessment of body volume status also in HD patients [19]. They have been shown not only to be useful to positively impact clinical outcomes such as overhydration or systolic blood pressure [20,21], but have also been proven useful as a supplement to clinical assessment of dry weight before hemodialysis [22]. However, routine BIS is both time-consuming and labor-intensive and cannot be used continuously throughout treatment. By contrast, modern HD systems continuously and noninvasively track relative blood volume (RBV, first described in 1998 [23]), defined as the change in circulating blood volume, as an integrated, low-overhead module. Within the Fresenius 5008 system, the integrated Blood Volume Monitor (BVM) estimates RBV by deriving total protein concentration (TPC) from ultrasound-based measurements of sound velocity in the arterial line, corrected for temperature [23]. TPC, reflecting plasma proteins and hemoglobin, is then converted into ΔRBV. Hematocrit and hemoglobin values are calculated from linear equations assuming a standard baseline protein concentration [24]. UF is automatically regulated according to a patient-specific critical RBV: The machine calculates a maximum UF rate as twice the prescribed UF divided by the remaining session time. The actual UF rate is scaled by a coefficient that begins at 1, remains constant until halfway to the critical RBV, then declines linearly. When the critical RBV is reached, the coefficient falls to zero, halting UF [25]. Despite this practicality, it remains uncertain—and has been questioned—whether intradialytic RBV behavior meaningfully reflects the pre-dialysis volume state or extracellular fluid expansion, beyond signaling intradialytic refilling capacity [26,27]. Of note, there are data that link RBV dynamics to dialysis mortality [28]. 

This study examines if the values obtained by BVM monitoring (that is, the RBV) during HD can also be used to estimate volume status prior to dialysis. In particular, we tested the hypothesis that the extent of the decrease in RBV in the early steady phase of a hemodialysis session correlates with pre-dialysis body water measured using bioimpedance. 

## 2. Materials and Methods

### 2.1. Study Characteristics and Ethics

The presented data were obtained as an ancillary study of the SkInDialysis trial. The study was designed as a monocentric, prospective, clinical study conducted at the University Hospital Marburg, Department of Internal Medicine, Division of Nephrology, Germany. Patients were eligible if they were treated with hemodialysis thrice a week with regular ultrafiltration exceeding 500 mL per session and if they were capable of reading, understanding, and signing the study information and informed consent form, respectively. Patients were excluded in case of acute illness (e.g., cardiac decompensation, infection) because of possible impact on fluid homeostasis and hemodynamic stability. The study protocol followed the Declaration of Helsinki. The study was approved by the Medical Faculties’ Ethics Committee of the Philipps University of Marburg (approval number: 24-236-BO, dated 16 October 2024) and is registered in the German Clinical Trials Register (DRKS, ID: DRKS00036332; registration date 10 March 2025). Every patient gave written informed consent. The aim of the SkInDialysis study was to characterize adaptive changes in skin water loss/conservation during forced and pronounced intravascular water loss via ultrafiltration. In the SkInDialysis study we investigated changes in water handling by the skin via measurement of transepidermal water loss, evaporative cooling, and heat diffusion using the Tewameter (Courage + Khazaka Electronic Ltd, Cologne, Germany). In the study presented here we asked whether RBV dynamics are mirrored by changes in water loss as assessed by body composition and ultrafiltration. All patients were treated using the same dialysis machine (Fresenius 5008 CorDiax, Fresenius, Bad Homburg, Germany). Ultrafiltration and RBV were measured by the dialysis machine. 

### 2.2. Bioimpedance Spectroscopy (BIS) Measurement Protocol

All participants were adult ambulatory patients receiving maintenance HD at the University Hospital Marburg. Measurements were performed on three consecutive dialysis sessions within one week (Monday–Wednesday–Friday or Tuesday–Thursday–Saturday) at five standardized time points: immediately before dialysis (T0), 20 min after initiation (T1), 80 min after initiation (T2), 160 min after initiation (T3), and immediately post-dialysis (T4). The participants were placed in a supine position and allowed a 5 min rest period before the first measurement to ensure accurate bioelectrical impedance readings.

All bioimpedance measurements were taken using the Bodystat Quadscan 4000 device (Bodystat Ltd.; London, UK). The electrodes required for bioimpedance assessment were placed on the hand (one electrode at the level of the distal metacarpal bones, the second at the midpoint of the wrist) and on the ipsilateral foot (one electrode at the level of the distal metatarsal bones, the second at the malleolus). Electrodes were placed on the side contralateral to the vascular access or central venous catheter and replaced after each measurement. We obtained the following parameters in every measurement: Total body water (TBW; percentage and absolute volume), extracellular water (ECW; percentage and volume), and intracellular water (ICW; percentage and volume). Body weight was measured immediately prior to dialysis using a calibrated scale (MPE 250K100PM, Kern & Sohn GmbH, Balingen, Germany). This weight served as the baseline value for T0. Laboratory parameters were obtained from the patient records of the laboratory system (LAURIS Client, Nexus AG, Donaueschingen, Germany). 

### 2.3. Statistical Analysis

We used GraphPad Prism software (version 10; GraphPad Software Inc., San Diego, CA, USA) for statistical analysis. Normality was assessed with the D’Agostino–Pearson test. For graphical display, individual values or mean ± standard error of the mean (S.E.M.) are shown. Descriptive statistics are reported as means with standard deviations (SD) or as absolute numbers with corresponding percentages. Group comparisons were performed using one-way ANOVA for independent groups or mixed-effects models for repeated-measures designs, followed in each case by Tukey’s post hoc test for multiple comparisons. Correlations were assessed using Pearson’s r as a measure of the strength and direction of an association. Nevertheless, in the body of the manuscript, “R^2”^ is stated because it reflects a more accessible measure of association, that is, “variance explained”. Statistical significance was defined as a *p*-value of < 0.05, with significance levels indicated as follows: * *p* < 0.05, ** *p* < 0.01, *** *p* < 0.001, **** *p* < 0.0001.

## 3. Results

### 3.1. Hemodialysis-Induced Changes in Body Fluid Compartments

**Figure 1:**
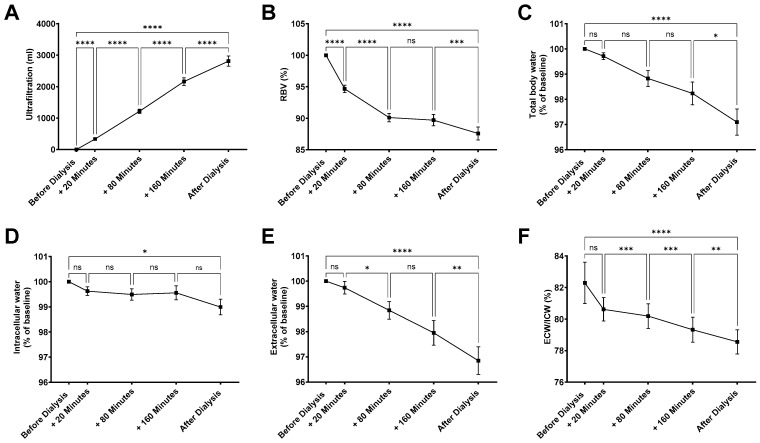
**Intradialytic BIS-derived body water measurements provide physiologically plausible values.** (**A**) Ultrafiltration (n = 31), (**B**) relative blood volume (RBV, n = 28), (**C**) total body water (n = 29), (**D**) intracellular water (n = 29), (**E**) extracellular water (n = 29), and (**F**) extracellular-to-intracellular water ratio (ECW/ICW, n = 29) were measured at the following time points: before HD, +20 min, +80 min, +160 min after the start of HD, and immediately after HD. Statistical analysis was performed using one-way ANOVA (**A**,**B**) or mixed-effects analysis (**C**–**F**) with Tukey’s multiple comparisons test, where applicable. Summary data are presented as mean ± SEM. * *p* < 0.05, ** *p* < 0.01, *** *p* < 0.001,**** *p* < 0.0001, ns = not significant. Of note, missing data were omitted.

In this study, 11 patients were examined (8 male/3 female) with a mean age of 55 ± 13 years. Patients were on hemodialysis for 106 ± 97.3 weeks. HD access was obtained with a central venous catheter in five patients and an AV fistula in six patients. All patients were Caucasian. Causes of end-stage kidney disease included diabetes (n = 2), IgA glomerulonephritis (n = 2), unspecified glomerulopathies (n = 2), malignancy (n = 2), congenital anomalies (n = 1), toxic kidney injury (n = 1), and multi-organ failure (n = 1). All patients completed three standardized dialysis sessions with continuous monitoring of ultrafiltration (UF), relative blood volume (RBV), and bioimpedance spectroscopy. In total, 31 measurements have been recorded for ultrafiltration, 28 measurements for RBV, and 29 measurements for BIS. In the studied cohort, total ultrafiltration volume increased linearly over the course of hemodialysis, with a mean total ultrafiltration of 2809 ± 894.2 mL per session (Figure 1A). Within the first 20 min, an ultrafiltration of 336 ± 134 mL was obtained, which was paralleled by a significant reduction in RBV to 94.7 ± 3.1% of baseline (Figure 1B). Over the entire course of the treatment, RBV decreased to 87.6 ± 5.5%. Dialysis treatment reduced total body water (TBW) by 2.9 ± 2.7% (Figure 1C). Compartment-specific analyses revealed a distinct pattern: intracellular water (ICW) declined only modestly (−1.1 ± 1.65%), reaching statistical significance only when comparing pre- and post-dialysis measurements (Figure 1D). In contrast, ECW showed a more pronounced reduction (−3.15 ± 2.9%) (Figure 1E), already detectable during the treatment course. Consequently, the ECW/ICW ratio decreased significantly across dialysis (Figure 1F), reflecting preferential removal of extracellular volume. 

In summary, hemodialysis led to marked reductions in extracellular rather than intracellular water, leading to a progressive decline in RBV and the ECW/ICW ratio.

### 3.2. Correlation of UF with Changes in Body Water

**Figure 2:**
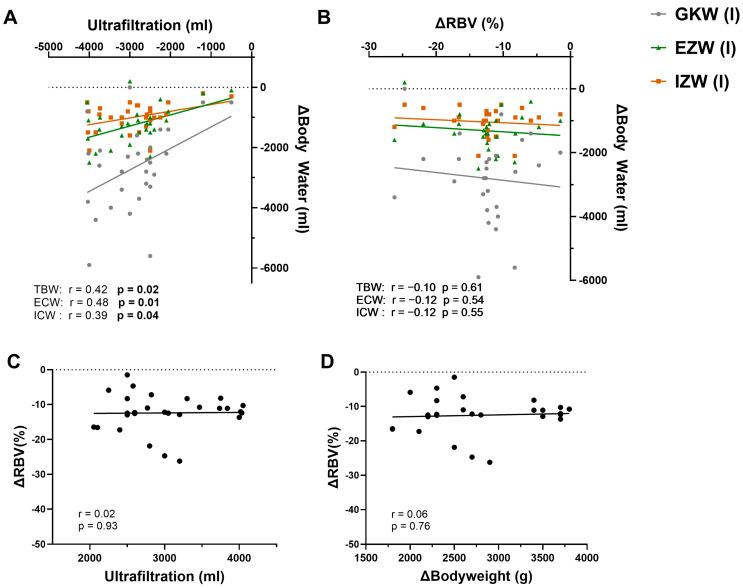
**Correlations between ultrafiltration, relative blood volume, and BIA-derived body water changes by one hemodialysis session.** (**A**) Correlation between ultrafiltration and changes (Δ) in body water compartments (intracellular water ICW, extracellular water ECW, and total body water TBW, n = 29). (**B**) Correlation between changes in relative blood volume (ΔRBV) and Δbody water (n = 27). (**C**) Correlation between ΔRBV and ultrafiltration (n = 28). (**D**) Correlation between ΔRBV and Δbody weight (n = 27). Measurements before HD and immediately after HD were included. Correlation analyses were performed using Pearson’s r.

We next assessed whether changes in body water composition during dialysis are reflected by ultrafiltration and RBV (Figure 2). Across all sessions, TBW reduction correlated significantly with cumulative UF (R^2^ = 0.18, *p* = 0.02; Figure 2A). When separated by compartments, the correlation was stronger for ECW (R^2^ = 0.23, *p* = 0.01) than for ICW (R^2^ = 0.16, *p* = 0.04), indicating that the extracellular compartment is significantly more affected by the removal of surplus water during HD than the intracellular compartment. By contrast, when comparing values before and after HD, changes in RBV (ΔRBV) showed no correlation with reductions in all measured parameters of body water: neither TBW (r = −0.10, *p* = 0.61), nor ECW (r = −0.12, *p* = 0.54), nor ICW (r = −0.12, *p* = 0.55) (Figure 2B) correlated with ΔRBV. Moreover, ΔRBV was also not correlated with the amount of UF achieved (Figure 2C) or the body weight reduction by HD (Figure 2D). 

Thus, while UF volume was proportionally reflected in compartmental body water loss, RBV decline was uncoupled from absolute fluid removal.

### 3.3. Early Intradialytic RBV Levels Reflect Pre-Dialysis Volume Status

**Figure 3:**
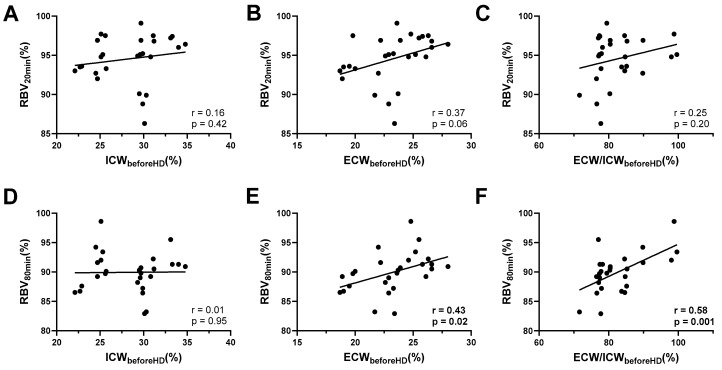
**Correlations between RBV at 20 and 80 min after HD initiation and BIA-derived body water parameters prior to HD.** (**A**–**C**) Correlation of RBV at 20 min with ICW, ECW, and the ECW/ICW ratio, respectively. (**D**–**F**) Correlation of RBV at 80 min with ICW, ECW, and the ECW/ICW ratio. Correlations were assessed using Pearson’s r.

To examine whether early RBV trajectories are informative for the pre-dialysis volume state, we related RBV values at 20 and 80 min to pre-dialysis bioimpedance parameters. RBV after 20 min was not significantly associated with pre-dialysis ICW (R^2^ = 0.03, *p* = 0.4) and only showed a nonsignificant trend towards correlation with ECW (R^2^ = 0.14, *p* = 0.06; Figure 3A–C). By contrast, RBV after 80 min demonstrated robust associations: while no relationship was observed with ICW (R^2^ = 0.01, *p* = 0.95), RBV at this time point correlated significantly with pre-dialysis ECW (R^2^ = 0.19, *p* = 0.02; Figure 3D) and even more strongly with the pre-dialysis ECW/ICW ratio (R^2^ = 0.34, *p* = 0.001; Figure 3E). 

Taken together, RBV trajectories during the early dialysis phase reflected the pre-dialysis extracellular volume status rather than the extent of ultrafiltration or body water removal.

## 4. Discussion

This study set out to determine whether device-derived RBV trajectories in the early phase of HD carry information about the pre-dialysis volume state as assessed by bioimpedance. Three principal observations emerged. First, fluid removal predominantly reduced ECW with only modest changes in ICW, yielding a lower ECW/ICW ratio over the session. Second, RBV decline was not proportional to the absolute amount of UF or body-water loss. Third, and most importantly, RBV levels in the early intradialytic window—particularly at ~80 min—were associated with pre-dialysis ECW and, more strongly, with the pre-dialysis ECW/ICW ratio, whereas RBV at 20 min showed only a non-significant trend. Together, these data suggest that early RBV dynamics reflect the size of the extracellular compartment present before HD rather than the magnitude of fluid removed during the treatment.

### 4.1. Plausibility of the Observed Signals (RBV, UF, Body Composition)

The internal consistency of the results is physiologically plausible. With net fluid removal from the intravascular space, capillary refill from the interstitium compensates for the fall in circulating volume; the magnitude and timing of this refill are determined by transcapillary Starling forces and the available interstitial reservoir—operationally the ECW compartment. In such a framework, (i) a stronger reduction in ECW than ICW during HD, (ii) an uncoupling between absolute UF and ΔRBV, and (iii) an association between RBV levels and pre-HD ECW/ICW in the steady state are coherent findings. The preference for ECW removal over ICW is well established in dialysis physiology and is mirrored here by the consistent fall in the ECW/ICW ratio. Conversely, ΔRBV—an index derived from hemoconcentration and plasma protein concentration—captures the interplay between vascular compliance and refill rather than the absolute volume removed; lack of correlation between ΔRBV and UF or Δbody water is therefore expected. That the association of RBV with pre-HD ECW strengthens by ~80 min (vs. 20 min) is also plausible: early measurement points are susceptible to transient non-steady-state phenomena (temperature shifts, initial redistribution, circuit priming effects), while a quasi-steady balance between UF and refill tends to emerge later within the first hour.

### 4.2. Relation to Existing Literature: What Is Known, What Is New

Our observations align with several strands of prior work. Bioimpedance spectroscopy (BIS) is validated for quantifying fluid compartments in HD patients [29] and has been associated with clinically relevant outcomes [19,30,31,32], with the caveat that its mortality impact remains debated. Mechanistically, HD preferentially mobilizes ECW, with relatively smaller and slower ICW changes. The Fresenius blood volume monitor (BVM) and related devices have been technically validated to track relative intravascular volume by inferring total protein concentration from ultrasound velocity; these systems have been leveraged in biofeedback paradigms targeting intradialytic hypotension through patient-specific RBV trajectories [33]. Against that background, our data confirm two established points and introduce one new element. Established points: (i) ECW predominates among the compartments affected by UF; (ii) ΔRBV is not a surrogate for absolute fluid removal [34]. The new element is the use of early RBV levels (rather than ΔRBV or end-session values) as an indirect window into the pre-HD extracellular volume state, with the 80 min time point offering the clearest signal. Prior studies have compared pre- and post-dialysis states or used RBV patterns to modulate UF in real time; by contrast, our analysis links the level of RBV during the early session to the pre-dialysis ECW/ICW ratio—an application that has received comparatively little attention. In parallel, we demonstrate feasibility and physiologic coherence of serial intradialytic BIS values under standardized conditions.

### 4.3. Alternative Explanations and Constraints

A key alternative explanation for the RBV–ECW association is that unmeasured factors (e.g., dialysate sodium, dialysate temperature, vascular access type, vasoactive medications, or baseline blood pressure) modify refill kinetics and thus RBV levels independently of ECW. While such factors could attenuate or confound the relationship, two observations argue that ECW size contributes materially: (i) the association strengthens as the session progresses into a more stationary phase (80 min), and (ii) the relationship is specific to ECW (and ECW/ICW) and absent for ICW. Another potential concern is measurement artifact—e.g., BVM assumptions about baseline protein concentration or BIS sensitivity during rapid fluid shifts. The congruence of findings across multiple, repeated time points (including a coherent compartment-specific pattern and expected correlations between UF and ECW changes) argues against gross artifact as a primary driver, though we acknowledge modest systematic biases are inherent to both technologies [35].

### 4.4. Implications for a Personalized Fluid-Management Strategy

Precision in fluid management depends on capturing both how much volume is present (compartment sizes) and how a given patient’s circulation tolerates removal (refill capacity and vascular reactivity). BIS informs the former (ECW/ICW), whereas RBV dynamics inform the latter (refill versus hemoconcentration). Our findings suggest a simple, device-integrated approach: using early RBV levels (e.g., around 60–90 min) as a low-overhead indicator of the pre-HD extracellular reservoir. A relatively higher RBV level at this stage—despite ongoing UF—may indicate a larger interstitial reserve and greater refill capacity, consistent with ECW expansion; conversely, a lower RBV level could flag limited refill. Pairing this real-time RBV signal with baseline BIS (and, where available, point-of-care ultrasound of lung or IVC) could inform individualized UF profiling (rate, profile shape), dialysate sodium or temperature choices, and early identification of sessions at risk for intradialytic hypotension. Although bioimpedance is considered to have “medium to high accuracy” to assess body volume, it surely is time- and personnel-intensive [9]. Our data now imply a link between in-device measurement of RBV with a bioimpedance-derived measure of volume status. By extension, the value of RBV as a measure of vascular compliance and refill at the time point 80 min after dialysis initiation can be complemented with additional information on volume load. Importantly, these are hypothesis-generating implications; prospective interventional testing is needed to determine whether such integrated, multimodal strategies translate into improved tolerance or outcomes. 

### 4.5. Limitations

Several limitations temper the inferences. The cohort is small and monocentric, with repeated sessions per patient; mixed-effects modeling would be preferable to account for within-patient correlation and to refine effect estimates. Nevertheless, the small sample size did not allow for meaningful further inference. Likewise, we did not systematically adjust for all potential modifiers of refill (e.g., dialysate sodium/temperature, medications, autonomic tone), and access type may influence hemodynamics. Both BIS and BVM have method-specific assumptions (e.g., BIS model parameters; BVM conversion from ultrasound velocity to total protein concentration and from protein concentration to hematocrit/hemoglobin) that may introduce small systematic biases without altering effect directionality. Finally, clinical end points (e.g., intradialytic hypotension, symptom burden, or hard outcomes) were not evaluated.

## 5. Conclusions

In a controlled, repeated-measures setting, we show that **early RBV levels during HD correlate with pre-dialysis ECW and ECW/ICW**, whereas RBV decline does not scale with absolute fluid removal. These findings are internally coherent, consistent with established physiology, and extend prior literature by positioning early RBV as a pragmatic, device-embedded indicator of the pre-dialysis extracellular reservoir. Future work should (i) validate these results in larger, diverse cohorts with mixed-effects and time-varying models; (ii) define clinically usable thresholds (e.g., ROC-based cut-offs for ECW expansion) and test integration with BIS and point-of-care ultrasound; and (iii) assess whether RBV-informed, multimodal personalization of UF and dialysate prescriptions improves intradialytic stability and patient-centered outcomes.

## Data Availability

All data are presented in the manuscript and its accompanying files.
